# Supplementary material for: Pharmacodynamics of Ceftriaxone, Ertapenem, Fosfomycin and Gentamicin in Neisseria gonorrhoeae
Source: Antibiotics (Basel). 2022 Feb 23;11(3):299. doi: 10.3390/antibiotics11030299 (PMC8944423; doi:10.3390/antibiotics11030299)
Supplement: Supplementary file 1 [file antibiotics-11-00299-s001.zip › antibiotics-1554942-supplementary.pdf]

Supplementary Materials

Table S1. Strain-per-strain comparisons of pharmacodynamic parameters for each antimicrobial

		Strain comparison (p-value)		
Antibiotic	Parameter	WHO F vs WHO X	WHO F vs CS03307	WHO X vs CS03307
CRO	ψ_{max}	0.23	0.31	0.35
	ψ_{min}	<0.0001	<0.0001	0.11
	κ	0.0169	<0.0001	0.0031
	zMIC	<0.0001	<0.0001	<0.0001
ERT	ψ_{max}	0.43	<0.0001	0.097
	ψ_{min}	<0.0001	<0.0001	0.0049
	κ	0.011	0.12	0.0013
	zMIC	<0.0001	<0.0001	<0.0001
FOS	ψ_{max}	0.64	0.23	0.12
	ψ_{min}	<0.0001	0.68	<0.0001
	κ	0.47	0.086	0.31
	zMIC	0.073	0.0001	<0.0001
GEN	ψ_{max}	0.81	0.28	0.32
	ψ_{min}	0.64	0.25	0.25
	κ	0.90	0.94	0.95
	zMIC	0.041	0.0039	0.84

P-values comparing parameters strain-per-strain are given above. Parameter estimates were obtained using a system of seemingly unrelated, non-linear regression models (Methods). Abbrieviations: CRO, ceftriaxone; ERT, ertapenem; FOS, fosfomycin; GEN, gentamicin.

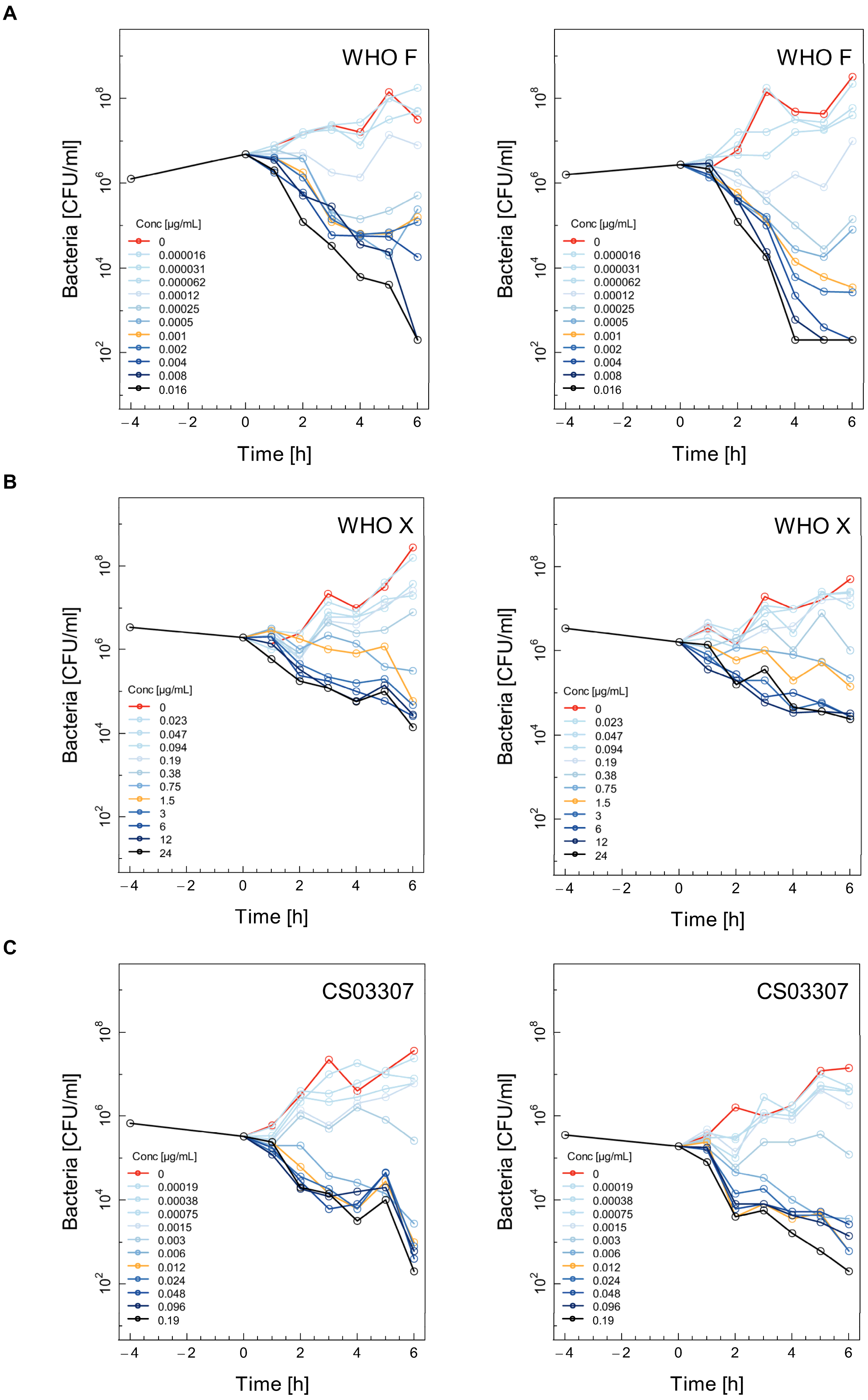

Supplementary Figure S1. Remaining time-kill curves (TKC) (i.e., the curves not shown in Figure 1) for three different strains of *Neisseria gonorrhoeae* using ceftriaxone. For each strain, TKC from two of the three independent experiments is shown: WHO F **(A)**, WHO X **(B)**, CS03307 **(C)**. For each figure, eleven doubling dilutions are plotted. The black line corresponds to the highest concentration of antibiotics used in the assay [16x the minimum inhibitory concentration (MIC)]. The yellow line represents the concentration corresponding to 1x MIC, while the red line represents growth in the absence of antimicrobials. The number of colony forming units (CFU)/ml was measured from 4 hours before until 6 hours after the addition of antimicrobials. The limit of detection was 200 CFU/ml.

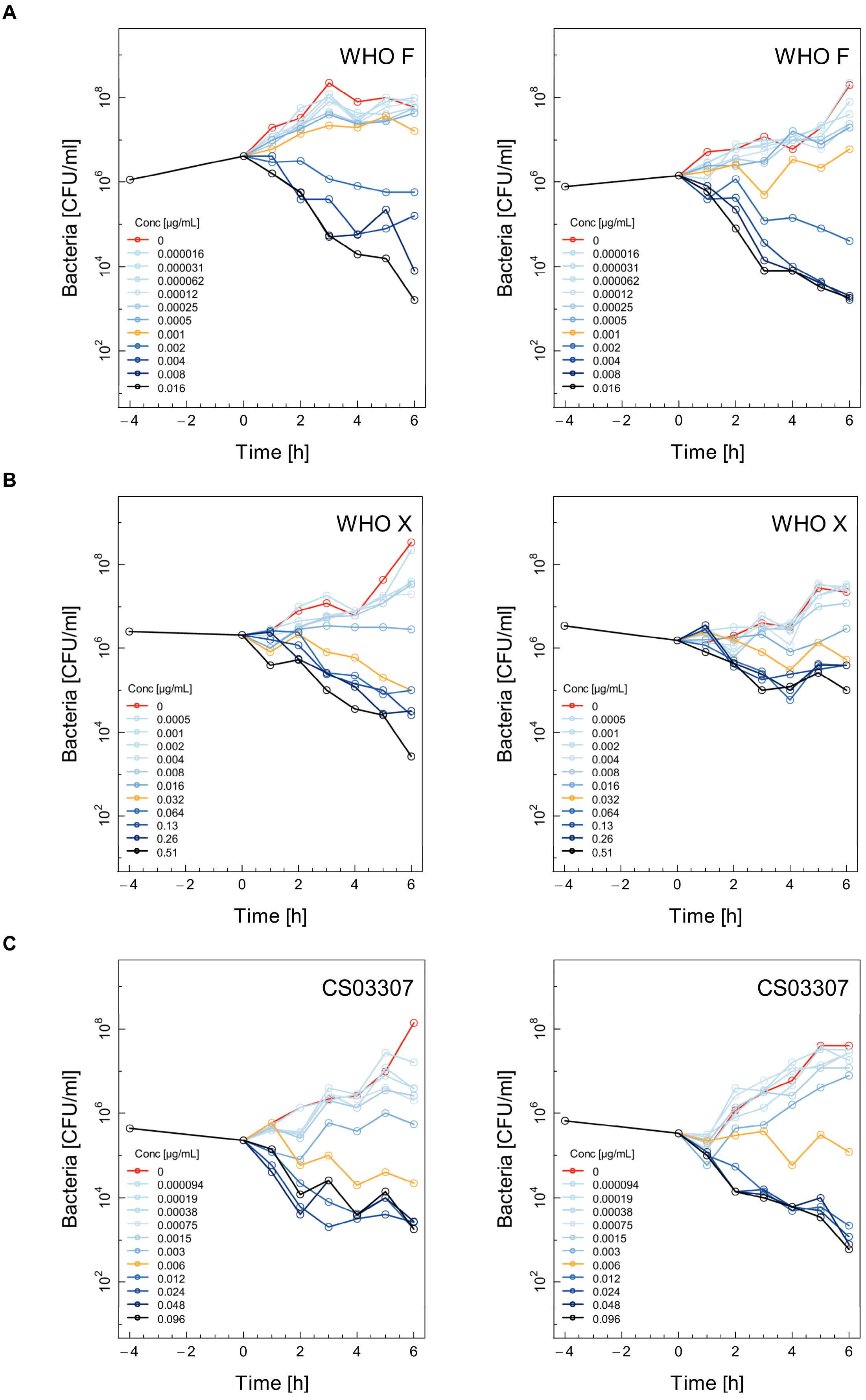

Supplementary Figure S2. Remaining time-kill curves (TKC) (i.e., the curves not shown in Figure 2) for three different strains of *Neisseria gonorrhoeae* using ertapenem. For each strain, TKC from two of the three independent experiments is shown: WHO F **(A)**, WHO X **(B)**, CS03307 **(C)**. For each figure, eleven doubling dilutions are plotted. The black line corresponds to the highest concentration of antibiotics used in the assay [16x the minimum inhibitory concentration (MIC)]. The yellow line represents the concentration corresponding to 1x MIC, while the red line represents growth in the absence of antimicrobials. The number of colony forming units (CFU)/ml was measured from 4 hours before until 6 hours after the addition of antimicrobials. The limit of detection was 200 CFU/ml.

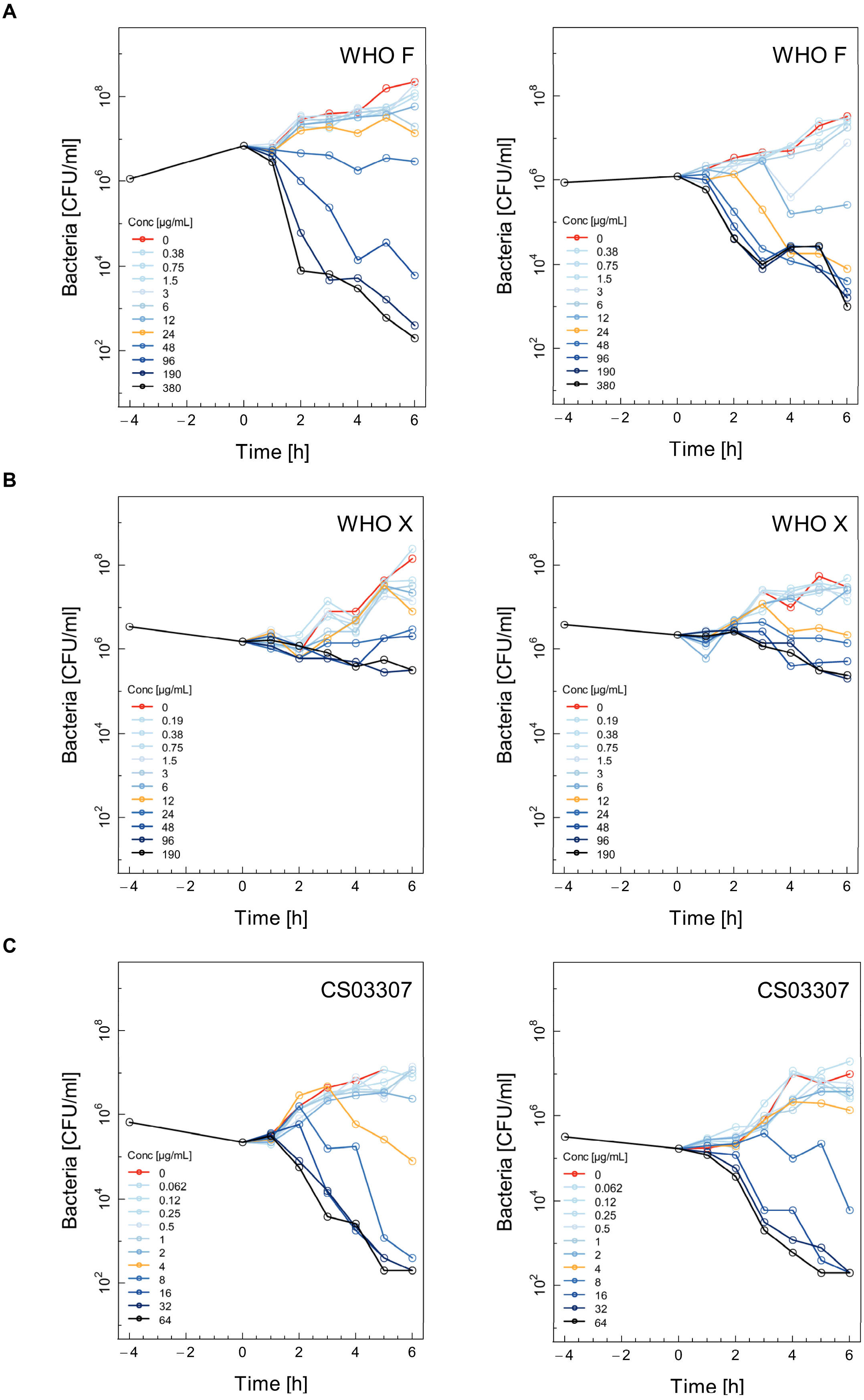

Supplementary Figure S3. Remaining time-kill curves (TKC) (i.e., the curves not shown in Figure 3) for three different strains of *Neisseria gonorrhoeae* using fosfomycin. For each strain, TKC from two of the three independent experiments is shown: WHO F **(A)**, WHO X **(B)**, CS03307 **(C)**. For each figure, eleven doubling dilutions are plotted. The black line corresponds to the highest concentration of antibiotics used in the assay [16x the minimum inhibitory concentration (MIC)]. The yellow line represents the concentration corresponding to 1x MIC, while the red line represents growth in the absence of antimicrobials. The number of colony forming units (CFU)/ml was measured from 4 hours before until 6 hours after the addition of antimicrobials. The limit of detection was 200 CFU/ml.

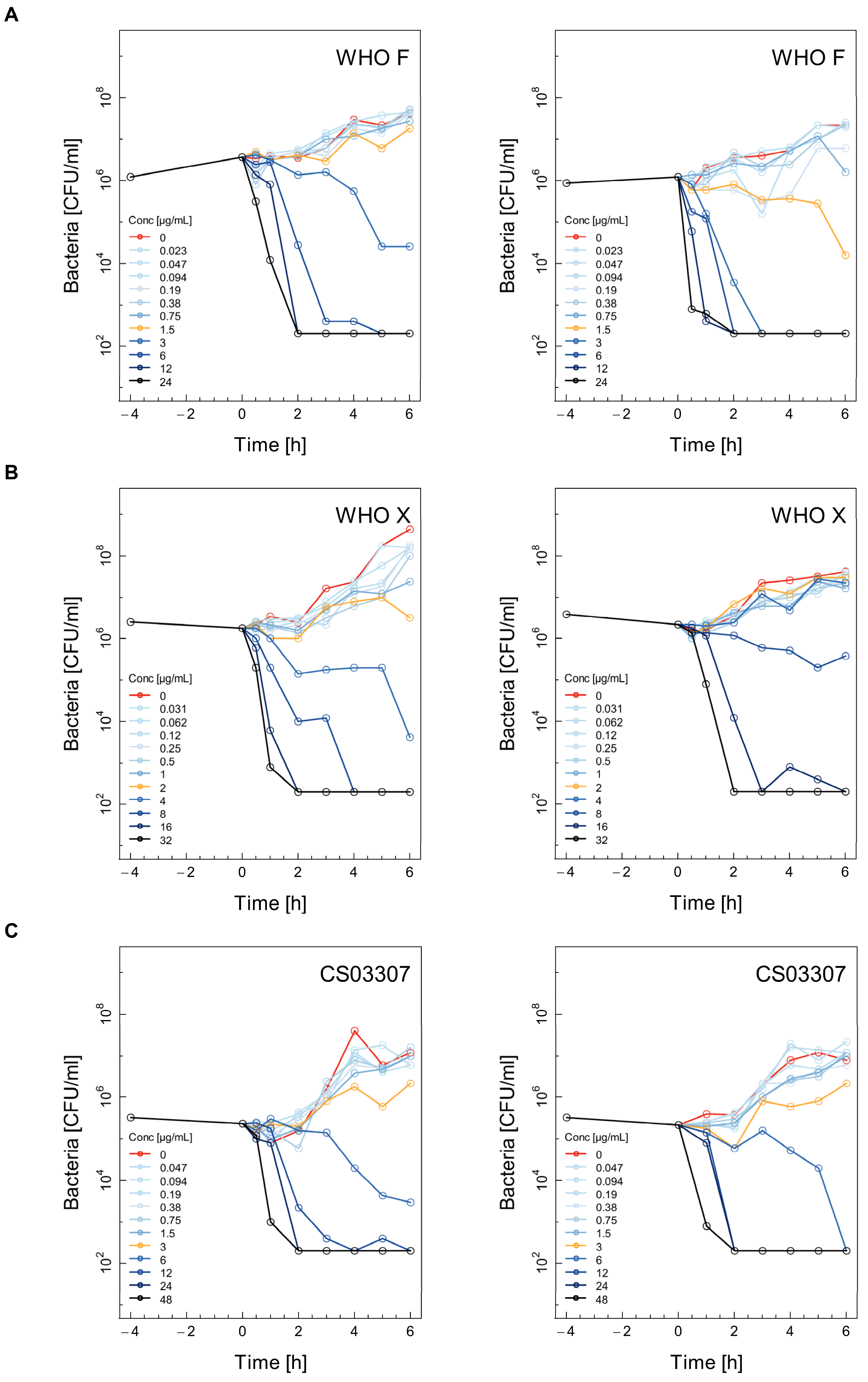

Supplementary Figure S4. Remaining time-kill curves (TKC) (i.e., the curves not shown in Figure 4) for three different strains of *Neisseria gonorrhoeae* using gentamicin. For each strain, TKC from two of the three independent experiments is shown: WHO F **(A)**, WHO X **(B)**, CS03307 **(C)**. For each figure, eleven doubling dilutions are plotted. The black line corresponds to the highest concentration of antibiotics used in the assay [16x the minimum inhibitory concentration (MIC)]. The yellow line represents the concentration corresponding to 1x MIC, while the red line represents growth in the absence of antimicrobials. The number of colony forming units (CFU)/ml was measured from 4 hours before until 6 hours after the addition of antimicrobials. The limit of detection was 200 CFU/ml.
